# Grand Challenges in Obstetrics and Gynecology

**DOI:** 10.3389/fmed.2022.939858

**Published:** 2022-06-29

**Authors:** Simcha Yagel, Sarah M. Cohen

**Affiliations:** Division of Obstetrics and Gynecology, Hadassah Medical Center, Faculty of Medicine, Hebrew University of Jerusalem, Jerusalem, Israel

**Keywords:** obstetrics, gynecology, outreach, preventative medicine, maternal care

The Obstetrics and Gynecology Specialty Section of Frontiers in Medicine will welcome submissions from the clinical, basic, and translational research spheres in all sub-specialties of obstetrics and gynecology. In addition to manuscripts reporting original research, we welcome meta-analyses and narrative or expert reviews exploring noteworthy topics of clinical care and research in OB/Gyne. Case reports and case series that report remarkable findings with potentially significant impact on clinical or research endeavors, will also be considered. The performance and dissemination of research is the key to advancing individual and population health and the practice of OB/Gyne worldwide.

Many challenges are on the radar for immediate and long-range research in OB/Gyne. The Great Obstetric Syndromes continue to pose short and long-term threats to the health and wellbeing of gravidae and their offspring. The last 2 years have brought into sharp focus the hazards of infectious disease in pregnancy. Hundreds of clinical and basic research papers have delved into the impact of SARS-CoV-2 infection on parturients and fetuses. However other pathogens remain dangerous in pregnancy, such as human cytomegalovirus. Work to develop an effective CMV vaccine continues. Other, preventable diseases may see a resurgence among populations with poor vaccination coverage. Going forward, research will examine the longer-term implications of COVID in pregnancy, as well as new strategies for prenatal surveillance of fetal CMV.

Artificial intelligence, with its myriad applications, has an essential role to play in the service of OB/Gyne. Clinical and basic researchers in all our subspecialties have only begun to scratch the surface of these modalities. AI and machine learning have been used to develop prediction algorithms for use in the labor and delivery ward, for example. AI has also been applied to improve diagnosis and prognostication in ovarian cancers, by incorporating and considering multiple factors. AI shows great promise in the field of OB/Gyne imaging more broadly, in image interpretation in fetal scanning for example. In the near future, AI will provide important assistance in the provision of care across many disciplines within our specialty.

One of the areas with potential to benefit significantly from AI applications is precision medicine. Via analysis of big data it will become increasingly feasible to tailor diagnostics and treatment to the individual patient, taking into consideration all aspects of their history, background and particular needs. The experience of the last few years has highlighted the urgent necessity and the possibilities of providing services to remote or poorly served regions and populations. Remote telehealth services blossomed in response to the demands of the pandemic: the approaches developed can serve in increasing access for marginalized patient groups. Creative solutions can be enlisted to provide access to reproductive care where access is constrained.

As physicians and researchers in OB/Gyne we might think of “Outreach” in the context of programs designed to reach geographically remote communities. However outreach programs can bring life-saving and quality of life enhancing services and professional training anywhere they are needed. Creative approaches combining local knowledge and expertise with professional resources are essential to providing customized care.

Prevention, whether of obstetric complications or STI's, congenital anomalies or ovarian cancer, is a fundamental part of OB/Gyne clinical and research endeavors. Preventative medicine and investigations can take the most advanced or basic approaches, the key being to devise interventions that are shown to be suited to target populations and produce sustainable results.

Within preventative medicine, the many factors that contribute to individual risks and outcomes must be considered. Innumerable clinical trials over the course of decades excluded female subjects and thereby failed to provide evidence-based research to inform clinical practice for female patients across all disciplines. The medical community has begun to grapple with this and many other sources of systemic prejudice and bias that permeate our profession. It is imperative to identify and overcome these entrenched biases in order to deliver optimal, personalized care to individual patients. Studies focusing on clinical training in OB/Gyne, healthcare delivery, patient perception, and medical and scientific communication, among many other topics, can lay the foundation on which to develop evidence-based approaches to remediation.

An indispensable part of work in OB/Gyne—in the clinic and the lab—is imaging. Studies reporting on or applying all methodologies and modalities of imaging, including ultrasound, MRI, CT, and others, as relates to our work in OB/Gyne, whether illustrating interesting cases or technical advances, contributing to clinical diagnosis, or showing how imaging is used in the service of basic research advancement, are welcome.

We are excited to take part in this specialty section in Obstetrics and Gynecology, creating a forum for sharing and dissemination of meaningful, impactful research. We look forward to receiving your submissions.

## Author Contributions

All authors listed have made a substantial, direct, and intellectual contribution to the work and approved it for publication.

## Conflict of Interest

The authors declare that the research was conducted in the absence of any commercial or financial relationships that could be construed as a potential conflict of interest.

## Publisher's Note

All claims expressed in this article are solely those of the authors and do not necessarily represent those of their affiliated organizations, or those of the publisher, the editors and the reviewers. Any product that may be evaluated in this article, or claim that may be made by its manufacturer, is not guaranteed or endorsed by the publisher.

